# Modelling the association between COVID-19 transmissibility and D614G substitution in SARS-CoV-2 spike protein: using the surveillance data in California as an example

**DOI:** 10.1186/s12976-021-00140-3

**Published:** 2021-03-09

**Authors:** Shi Zhao, Jingzhi Lou, Lirong Cao, Hong Zheng, Marc K. C. Chong, Zigui Chen, Benny C. Y. Zee, Paul K. S. Chan, Maggie H. Wang

**Affiliations:** 1 JC School of Public Health and Primary Care, Chinese University of Hong Kong, Hong Kong, China; 2 CUHK Shenzhen Research Institute, Shenzhen, China; 3 Department of Microbiology, Chinese University of Hong Kong, Hong Kong, China

**Keywords:** COVID-19, Spike protein, Mutation, Transmission, Statistical modeling

## Abstract

**Background:**

The COVID-19 pandemic poses a serious threat to global health, and pathogenic mutations are a major challenge to disease control. We developed a statistical framework to explore the association between molecular-level mutation activity of SARS-CoV-2 and population-level disease transmissibility of COVID-19.

**Methods:**

We estimated the instantaneous transmissibility of COVID-19 by using the time-varying reproduction number (*R*_*t*_). The mutation activity in SARS-CoV-2 is quantified empirically depending on (i) the prevalence of emerged amino acid substitutions and (ii) the frequency of these substitutions in the whole sequence. Using the likelihood-based approach, a statistical framework is developed to examine the association between mutation activity and *R*_*t*_. We adopted the COVID-19 surveillance data in California as an example for demonstration.

**Results:**

We found a significant positive association between population-level COVID-19 transmissibility and the D614G substitution on the SARS-CoV-2 spike protein. We estimate that a per 0.01 increase in the prevalence of glycine (G) on codon 614 is positively associated with a 0.49% (95% CI: 0.39 to 0.59) increase in *R*_*t*_, which explains 61% of the *R*_*t*_ variation after accounting for the control measures. We remark that the modeling framework can be extended to study other infectious pathogens.

**Conclusions:**

Our findings show a link between the molecular-level mutation activity of SARS-CoV-2 and population-level transmission of COVID-19 to provide further evidence for a positive association between the D614G substitution and *R*_*t*_. Future studies exploring the mechanism between SARS-CoV-2 mutations and COVID-19 infectivity are warranted.

**Supplementary Information:**

The online version contains supplementary material available at 10.1186/s12976-021-00140-3.

## Introduction

Coronavirus disease 2019 (COVID-19) caused by severe acute respiratory syndrome coronavirus 2 (SARS-CoV-2) was first reported in 2019 [1–5]. The COVID-19 pandemic poses a serious threat to global health and has spread to over 200 countries globally in a short period of time [6, 7]. In response to the ongoing COVID-19 pandemic, the World Health Organization (WHO) declared a public health emergency of international concern on January 30, 2020 [8]. As of September 6, 2020, over 27 million COVID-19 cases have been confirmed worldwide, with over 0.8 million deaths associated with COVID-19 [9].

The dynamics of the transmission of an infectious disease are largely determined by the pathogen’s infectiousness and the course of the transmission [10–12]. As a contagious disease with high transmissibility, the control of COVID-19 requires knowledge of the driving factors that may affect disease transmission [13–16]. Pathogenic mutations in SARS-CoV-2 are a major challenge for controlling COVID-19 [17, 18]. Early in February 2020, genetic variants with the D614G substitution on the SARS-CoV-2 spike (S) protein began to spread first in Europe [19] and globally and were suspected to potentially affect viral transmission [20]. Here, ‘D614G’ denotes the amino acid substitution that changes aspartic acid (D) to glycine (G) on codon 614 of the S protein of SARS-CoV-2. However, the evident relationship between the molecular-level mutation activity of SARS-CoV-2 and the population-level transmissibility of COVID-19 remains unrevealed.

It is biologically reasonable that mutations in viral genomes may alter the pathogenic profile in terms of viral fitness and functionality [21, 22] and consequently change its transmissibility. Previous literature about seasonal influenza epidemics [23] suggested that a few key amino acid substitutions may lead to remarkable changing dynamics of epidemiological outcomes at the population scale. In this study, we adopted a statistical framework to explore and examine the association between COVID-19 transmissibility and key mutation activities in the S protein of SARS-CoV-2.

## Data and methods

### SARS-CoV-2 sequencing data and COVID-19 surveillance data

The full-length human SARS-CoV-2 strains in California were collected via the Global Initiative on Sharing All Influenza Data (GISAID) [24] on May 24, 2020. A total of 524 strains were searched with collection dates ranging from January 22, 2020, to May 8, 2020. Table 1 summarizes the total number of strains in GISAID and the sample size included in this study for different periods. Since the number of sample stains varied by period, we set 9 successive periods and downloaded a stable number of strains for each period. In the period when more than 30 strains were available, we randomly sampled 30 strains. This sampling scheme is purposely designed to balance the weights due to different sample sizes that may affect the sliding window framework applied in quantifying the mutation activity (details in the next section). Sequences of all SARS-CoV-2 strains acquired are provided in the Additional file 1.

**Table 1:** Number of human SARS-CoV-2 strains in GISAID and the sample sizes included in this study for different periods

Period	Number of strains	
	in GISAID	in this study
Jan 1–31	6	6
Feb 1–29	16	16
Mar 1–10	72	30
Mar 11–20	94	30
Mar 21–31	158	30
Apr 1–10	100	30
Apr 11–20	21	21
Apr 21–30	50	30
May 1–10	7	7
Total	524	199

**Fig. 1:**
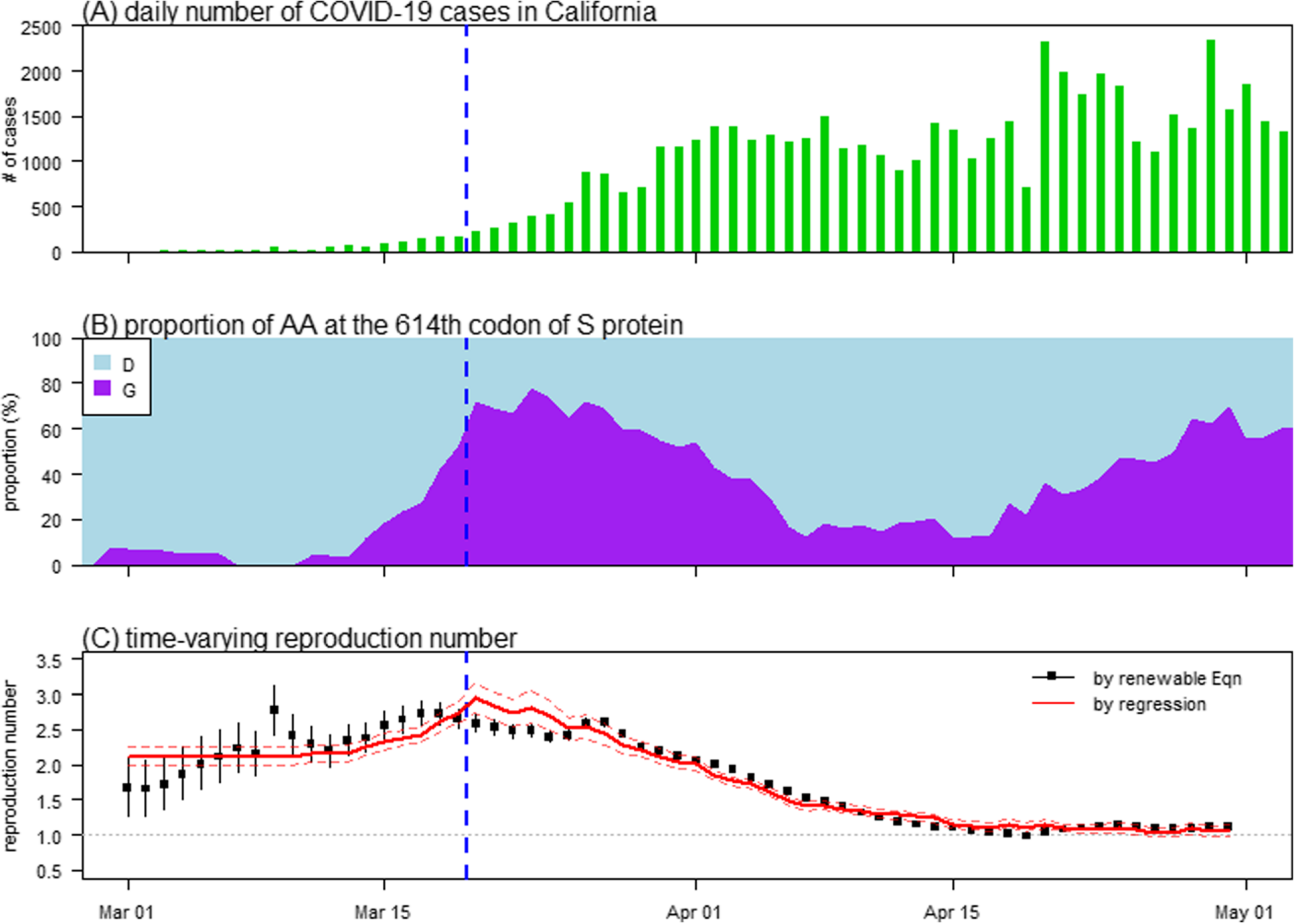
The number of COVID-19 cases (panel A), prevalence of the amino acids (AA) on the 614-th codon of the S protein (panel B), and time-varying reproduction number (*R*_*t*_, panel C) in California from March to April 2020. Panel A shows the daily number of COVID-19 time series. Panel B shows the prevalence of the AAs, including Aspartic Acid (D, in cyan) and Glycine (G, in purple), on the 614-th codon of the S protein. Panel C shows the *R*_*t*_ estimated (black) from the number of cases data using renewable equation and fitted (red) by using the mutation activity on the 614-th codon of the S protein in panel B. In each panel, the vertical dashed blue line represents the date, March 19, when the ‘stay-at-home’ order was officially implemented in California

Multiple sequence alignment was performed using Clustal Omega (accessed via https://www.ebi.ac.uk/Tools/msa/clustalo/), and the SARS-CoV-2 strain ‘*China/Wuhan-Hu-1/2019|EPI_ISL_402125*’ was considered as the reference sequence. The surveillance data of the daily number of COVID-19 cases in California were collected from the **R** package “*nCov2019*” [25] and The New York Times, accessed via https://github.com/nytimes/covid-19-data and https://www.nytimes.com/interactive/2020/us/coronavirus-us-cases.html, respectively. Figure 1a shows the daily number of COVID-19 cases in California in a time series.

### Instantaneous reproduction number and study period

We adopted the time-varying reproduction number (*R*_*t*_) to quantify the instantaneous COVID-19 transmissibility in California. Using the framework in [26], we estimated the time-varying reproduction number (*R*_*t*_) to quantify the instantaneous transmissibility of COVID-19 in California. Following the estimation framework developed in previous studies [26, 27], the epidemic growth of COVID-19 was modeled as a branching process, and thus, *R*_*t*_ can be expressed by using the renewable equation as follows:

$$ R(t)=\frac{C(t)}{\int_0^{\infty }w(k)C\left(t-k\right)\mathrm{d}k}, $$

where *C*(*t*) is the number of new COVID-19 cases reported at the *t*-th date. The function *w*(∙) is the distribution of the generation time (GT) of COVID-19. By averaging the GT estimates from the existing literature [28–35], we considered *w* as a Gamma distribution with a mean (±SD) value of 5.3 (±2.1) days. Slight variations in the settings of the GT did not affect our main findings.

For the selection of the study period, we considered both the quality of datasets and the increasing intensity (or effects) of local control measures. The selected study period for the COVID-19 surveillance data in California was from March 1, 2020, to April 30, 2020. During this study period, local COVID-19 surveillance was already following the governmental protocol, and the composition of disease control measures was relatively simple and adjustable in further multivariate analyses. In particular, an official ‘stay-at-home’ order was issued on (*t*_0_ =) March 19, 2020, in California (see https://covid19.ca.gov/stay-home-except-for-essential-needs/), which may affect the patterns of *R*_*t*_. Hence, we accounted for the effect of this local control measure in further multivariate analyses.

Our analyses depended on both (i) the quality of the data and (ii) the effects of the covariates, especially public health control measures that may decrease *R*_*t*_. Thus, one of the other reasons, which limited us to consider time outside the study period from March 1, 2020, to April 30, 2020, is related to the prevalence of mutation activities in SARS-CoV-2. During this study period, D614G appears to be the only major amino acid (AA) substitution in the S protein. Thus, complex interactive effects of multiple mutations on infectivity are less likely. As such, our analysis is simplified and is restricted in examining the effect of a single AA substitution.

### Quantifying the time-varying molecular-level mutation activity

In previous studies [36–38], a statistical framework was proposed to quantify genetic mutation activities associated with population-level outbreak situations by a metric, namely, the g-measure, on a real-time basis. The g-measure is an empirical time-varying metric calculated from the sequencing data of the pathogen and is determined by a predefined dominance prevalence threshold, *θ*, ranging from 0 to 1. The *θ* is the mutation prevalence threshold above which a molecular-level mutation (or substitution) is considered to affect the changing dynamics of the outbreak situation at the population level. The g-measure quantifies the level of key substitutions on a real-time basis, which allows one to explore its linkage to other time-varying variables [39].

We calculated the daily prevalence of amino acids (AA) on each codon in the S protein of SARS-CoV-2. We use *p*_*ij*_(*t*) to denote the prevalence of the *i*-th type of amino acid (AA) on the *j*-th codon of the S protein at time (or date) *t*, for *i* = 1, 2, …, 20, *j* = 1, 2, …, 1273, and *t* ranging from January 22 to May 8, 2020. Then, for each AA (20 in total) on each codon (1273 in total) of the S protein, we empirically calculated the prevalence time series. A sliding window was applied to the whole study period, from January 22, 2020, to May 8, 2020, to address the problem of the insufficient daily sample size. Let *W* denote the window size that represents a constant period (e.g., one week or one month). Hence, for *p*_*ij*_(*t*) on date *t*, we accounted for the proportion of the *i*-th AA out of all 20 types of AAs on the *j*-th codon within the time period of *t* ± *W*/2. In this study, we set *W* at 7 days for convenience, and we concluded that a variation in *W* did not affect our main results.

This sliding window scheme requires that the daily sample sizes of sequencing data are close in scale [38]. This guarantees that the prevalence series can reveal the real-world changing patterns of the mutation activity rather than bias towards a particular period with a large number of sequencing samples. Otherwise, as a simple example, during the periods before or after date *t*, i.e., from *t* − *W*/2 to *t* and from *t* to *t* + *W*/2, the prevalence may approach one period with a larger sample size.

**Fig. 2:**
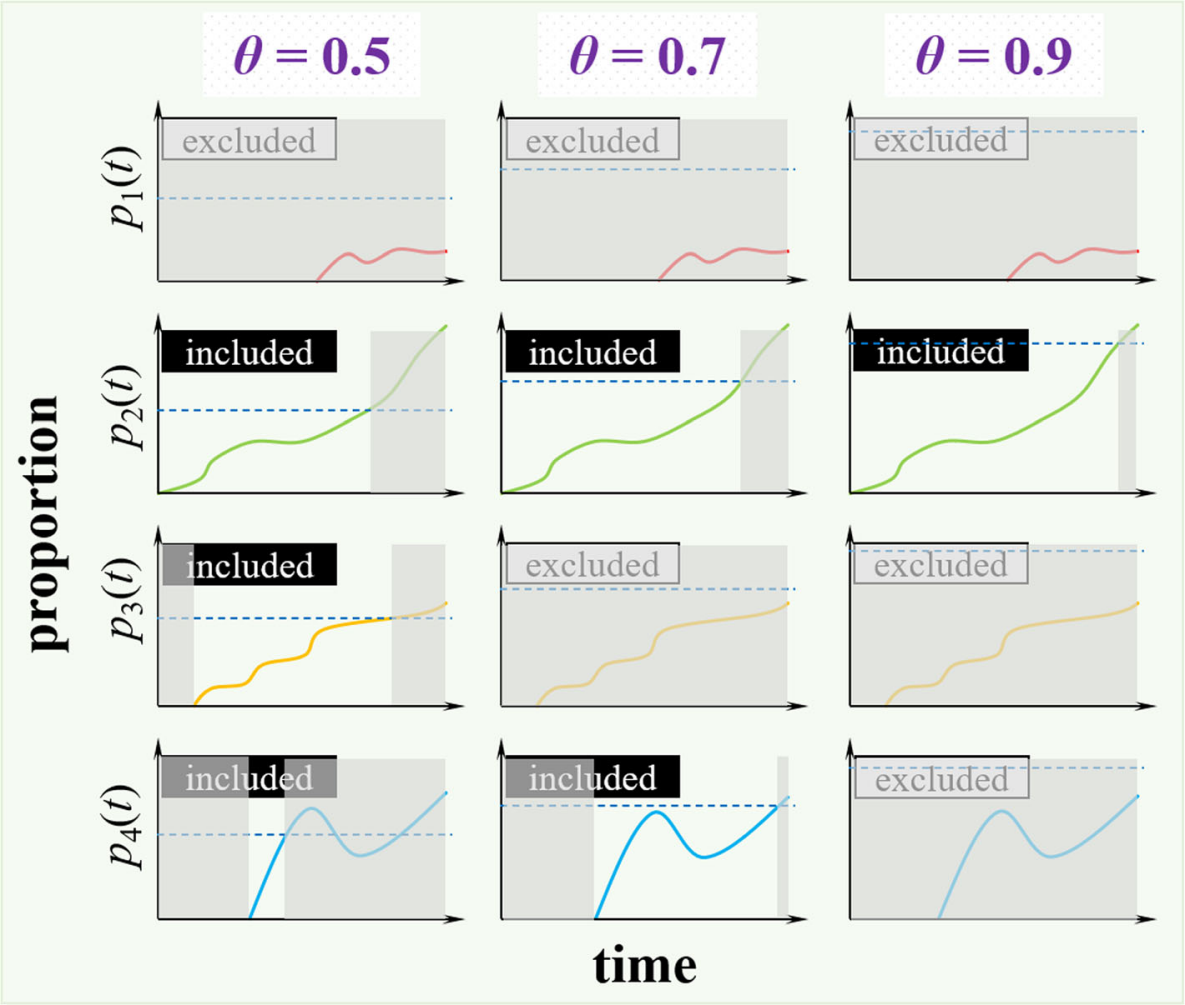
Illustration diagram of the analytical procedure of g-measure calculation. The prevalence time series of 4 different AA substitutions are denoted by *p*_1_(*t*), *p*_2_(*t*), *p*_3_(*t*) and *p*_4_(*t*), and indicated in red, green, orange and blue, respectively. Three scenarios of dominant prevalence threshold parameter, *θ*, are demonstrated with *θ* = 0.5, 0.7, and 0.9, respectively, which is indicated by the horizontal dashed line in each panel. The g-measure counts the segments of the prevalence series that start from 0 and increase over *θ*. In each panel, the prevalence series that accounts for g-measure is not shaded in grey region. In other words, the shaded regions are those part of prevalence series excluded from the g-measure calculation. For those prevalence series that never excess *θ*, they are excluded from g-measure calculation as labeled by ‘excluded’; otherwise, ‘included’ label is indicated

Following the calculations from previous studies [36, 37, 39], the g-measure counts the segments of the prevalence series that start from 0 and increase and eventually hits the level of *θ*. Prevalence series that never excess *θ* are excluded from the g-measure calculation. For other prevalence series that excess *θ* at some time point, only those parts start from 0 and increase and hit the level of *θ* are included in the g-measure calculation. An illustration diagram of the g-measure calculation is presented in Fig. 2. Technically, the algorithm in Table 2 is used to find the indicator function, *I*(*t*), to identify the segments of the prevalence series for the g-measure calculation. Therefore, given *θ*, the g-measure on date *t*, denoted by g-measure_*t*_(*θ*), can be calculated as follows:

1 $$ {\mathrm{gmeasure}}_t\left(\theta \right)=\sum \limits_j\sum \limits_i{p}_{ijt}\bullet {I}_{ijt}\left(\theta \right) $$

**Table 2:** Algorithm of g-measure indicator function, *I*(*t*)

**input**: discretized prevalence time series, *p*_1:*T*_; dominance prevalence threshold, *θ* (> 0). **initialization**: parameter for recoding the zero-prevalence time point, *ξ* = 1, parameter for recoding excess time point, *σ* = 0, *I*_1:*T*_ = **0**. **for** *t* in 1:*T* **do** If *p*_*t*_ == 0, set *ξ* = *t*. If (*p*_*t*_ ≥ *θ* & *ξ* > *σ*), *I*_(*ξ* + 1):(*t* − 1)_ = **1**, *σ* = *t*. **end for** **output**: discretized indicator time series, *I*_1:*T*_.	

The g-measure quantifies genetic mutation activities and is used to explore the association with *R*_*t*_. The parameter *θ* is estimated with the likelihood framework that will be introduced in the remaining parts.

**Fig. 3:**
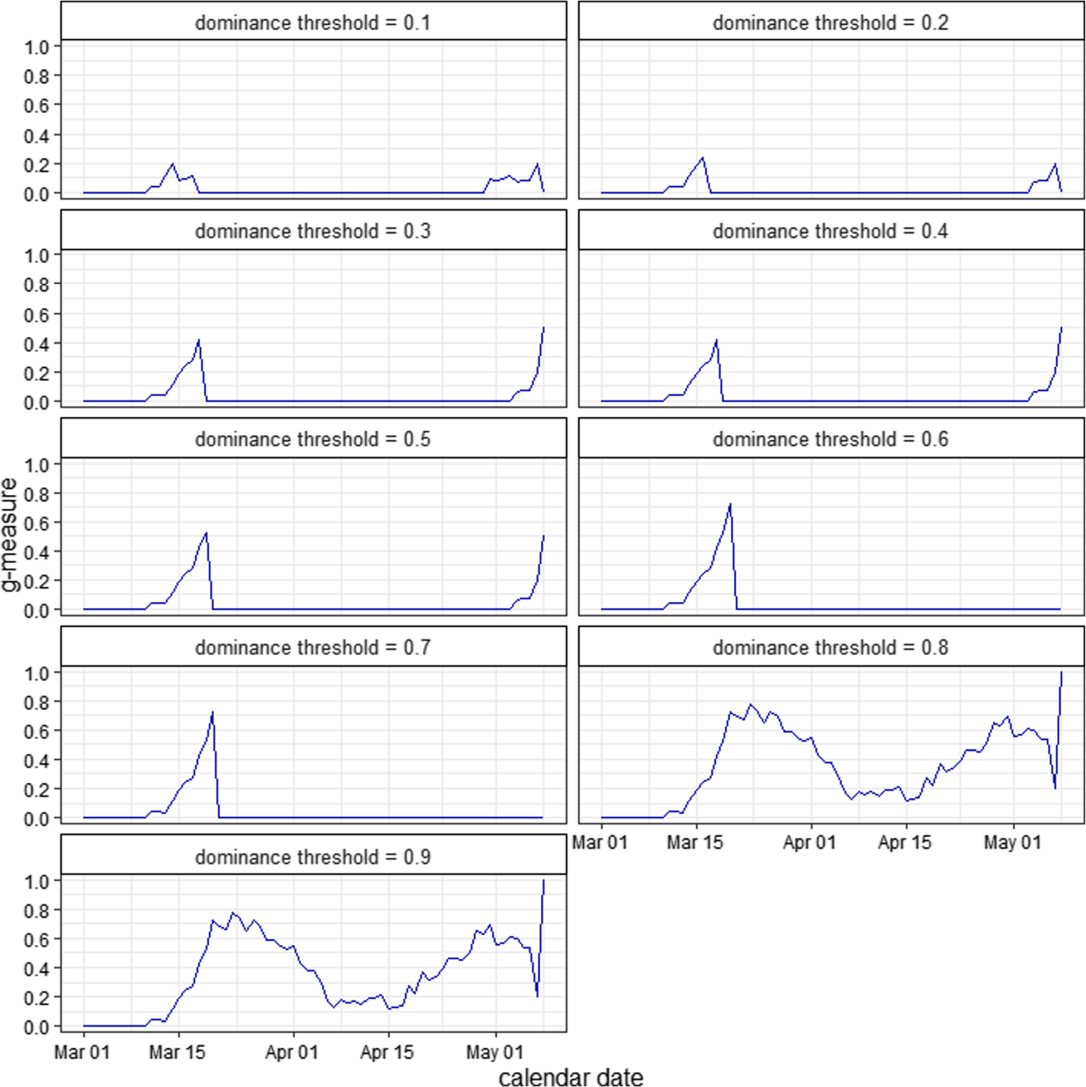
The g-measure time series of the SARS-CoV-2 spike (S) protein with different values of dominance threshold (*θ*) using Eqn (1)

Figure 3 shows the g-measure time series of the S protein with different values of *θ*. Note that only the g-measure time series from March 1, 2020, to April 30, 2020, were used in further regression analyses.

### Regression model and estimation of dominance prevalence threshold

We intended to explore the association between *R*_*t*_ and the mutation activity (measured by the g-measure) on the S protein. A multivariable regression model was fitted to examine the association between *R*_*t*_ and the g-measure considering the effect of local control measures in California.

Since *R*_*t*_ may be affected by disease control measures, we included a dummy variable with a discontinuity design to govern the effect of local control measures. In particular, the official ‘stay-at-home’ order was issued in California on March 19, 2020 (see https://covid19.ca.gov/stay-home-except-for-essential-needs/#stay-home-order). Hence, in the generalized linear regression model with discontinuity design, we set the structural break in the trends of *R*_*t*_ on March 19, 2020, which was denoted as *t*_0_. In previous studies, *R*_*t*_ is commonly modeled as a Gamma process [26, 40, 41], and thus, the regression is formulated in Eqn (2).

2 $$ \mathbf{E}\left[\ln \left({R}_t\right)\right]=c+a\;{\mathrm{gmeasure}}_t+b\;\mathbf{I}\left(t>{t}_0\right)\;\left(t-{t}_0\right) $$

Here, **E**[∙] is the function of the expectation. **I**(∙) is an indicator function that uses the binary variable (0 or 1);if variable *t* is larger than the threshold value *t*_0_, then 1; otherwise, it is 0. *c* is the constant parameter, and *a* and *b* are the slope parameters. Again, we fixed the term *t*_0_ to be March 19, 2020. The percentage change rate (*η*) of *R*_*t*_ associated with a 0.01 increase in the g-measure can be calculated directly from the slope parameter *a*. Thus, the term *η* is the effect size to be estimated of mutation activity on COVID-19 transmission, and we have *η* = [exp(*a* × 0.01) – 1] × 100%.

Following previous studies [40, 41], we considered *R*_*t*_ to follow a Gamma process with both means *R*_*t*_ and SDs *v*_*t*_ determined by the renewable equation. For a given time *t*, the Gamma distribution is denoted by *h*(∙|*R*_*t*_, *v*_*t*_), and we model exp. [*c* + *a*∙gmeasure_*t*_ + *b*∙**I**(*t* > *t*_0_)∙(*t* − *t*_0_)], which is the exponential of the right-hand side of Eqn (2), following the distribution *h*(∙|*R*_*t*_, *v*_*t*_). Thus, *h*(∙|*R*_*t*_, *v*_*t*_) is a function of parameters *a* and *θ* in Eqns (1) and (2), respectively, i.e., *h*(*a*, *θ* | *R*_*t*_, *v*_*t*_). In other words, both *R*_*t*_ and *v*_*t*_ were reconstructed directly from the number of cases in a time series (i.e., the raw data) and then served as the known parameters in the likelihood function *L*, which is given as follows:

$$ L\left(a,\theta \right)={\prod}_th\left(a,\theta |{R}_t,{v}_t\right). $$

The dominance prevalence threshold parameters *θ* and *a*, and equivalently *η*, can be estimated based on this likelihood framework and the regression model. Then, we calculated the maximum likelihood estimation (MLE) of *θ* to determine the g-measure for regression analysis. Using the likelihood framework, we estimated the MLE of the dominance prevalence threshold parameter *θ*, which was adopted to determine the g-measure and to examine the association with *R*_*t*_. The 95% CIs of the regression parameters were estimated by their point estimates plus or minus Student’s *t* distributed quantile multiplied by their standard errors. Since *η* and *a* are one-to-one mappings, the 95% CI of *η* can also be directly calculated from the 95% CI of *a*.

We employed Efron’s pseudo R-squared and likelihood-based partial R-squared to evaluate the goodness-of-fit of the regression model. A likelihood-ratio (LR) test on the scenarios with (as the full model) and without (as the baseline model) the g-measure was used to examine the reasonability of the model structure.

### Sensitivity analysis

Sensitivity analysis was carried out on the robustness and significance of the association between *R*_*t*_ and mutation activity. We conducted a sensitivity check on the effect size of mutation activity on the S protein in association with the changing dynamics of COVID-19 transmissibility in terms of the reconstructed *R*_*t*_. We considered three alternative regression formulas, which are similar to the main model Eqn (2), as follows:

3 $$ \mathbf{E}\left[\ln \left({R}_t\right)\right]=c+a\;{\mathrm{gmeasure}}_t $$

4 $$ \mathbf{E}\left[\ln \left({R}_t\right)\right]=c+a\;{\mathrm{gmeasure}}_t+b\;\mathbf{I}\left(t>{t}_0\right) $$

5 $$ \mathbf{E}\left[\ln \left({R}_t\right)\right]=c+a\;{\mathrm{gmeasure}}_t+b\;\mathbf{I}\left(t>{t}_0\right)\;\left(t-{t}_0\right)+d\;\mathbf{I}\left(t\le {t}_0\right)\;\left(t-{t}_0\right) $$

To check the robustness and significance of the estimates, we examined the consistency of both the sign (i.e., + or −) and the statistical significance (in terms of *p*-value < 0.05) of the regression coefficient *a* in the four models in Eq. (2)–(5).

## Results and discussion

We reconstructed the daily instantaneous reproduction number (*R*_*t*_) from the epidemic curve, as shown in Fig. 1c (black dots). We observed that the overall trends of *R*_*t*_ were relatively steady in the first half of March but gradually decrease thereafter since the local ‘stay-at-home’ order was issued in California on March 19, 2020, which was adjusted in Eqn (2). During the first half of March, which was regarded as the early phase of the outbreak, the reproduction number ranged from 1.5 to 3, and this range is generally consistent with previous estimates [2, 3, 6, 12, 29, 33, 42–48].

**Fig. 4:**
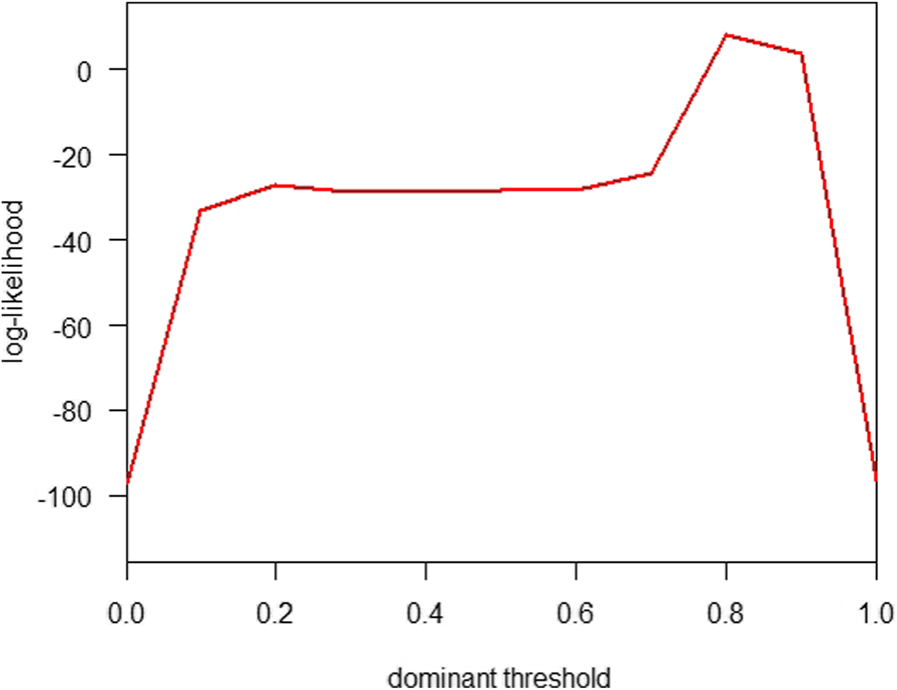
The likelihood profile of the dominant prevalence threshold parameter, *θ*, using the likelihood framework associated with regression model in Eqn (2)

We estimated the dominance prevalence threshold (*θ*) at 0.8, as shown in Fig. 4, which was adopted to examine the association between the g-measure and *R*_*t*_. When *θ* = 0.8, we found that the g-measure of the S protein appeared to be solely contributed to by the D614G substitution (see Fig. 1b), which also holds for all *θ* values > 0.75. In other words, the D614G substitution is considered a key mutation and is likely dominant in accounting for the changes in COVID-19 transmissibility due to a mutation at the molecular level.

Using the regression model in Eqn (2), we found a significant positive association between the g-measure and *R*_*t*_ when *θ* = 0.8 (as estimated). Hence, the changing dynamics of *R*_*t*_ are likely associated with the key mutations that are solely contributed to by the D614G substitution. We estimated that each 0.01 increase in the prevalence of glycine (G) on codon 614 is positively associated with a 0.49% (95% CI: 0.39 to 0.59) increase in *R*_*t*_, which, in terms of the partial R-squared, explains 61% of the *R*_*t*_ variation after accounting for the control measures. Figure 1c shows the fitting results by using the regression model in Eqn (2). By examining the patterns in Fig. 1, we found that the prevalence of the D614G substitution matches the trends of *R*_*t*_ in March 2020. However, we noticed that since (roughly) April 15, 2020, the prevalence of the D614G substitution increased, but *R*_*t*_ remained constant. The reasons may include that the increase in transmissibility was counteracted by the effects of local nonpharmaceutical interventions that reduced the transmission of COVID-19. Sensitivity analysis with alternative model structures in Eqns (3)–(5) indicates that the positive association between the D614G substitution and *R*_*t*_ holds robustly and significantly (data not shown).

The significant positive association between the D614G substitution and *R*_*t*_ is biologically reasonable and consistent with findings in previous studies. The few (but key) AA substitutions may vary the three-dimensional structure of the protein as well as influence the receptor binding process in which a pathogen invades host cells. Previous analysis implied that the D614G substitution may alter the conformation of the S protein and thus may theoretically functionally enhance receptor binding capacity [19, 20, 49, 50], leading to an increase in SARS-CoV-2 transmissibility and pathogenicity [51]. Similarly, we learn from the influenza virus that major antigenic changes can be caused by a single AA substitution related to the receptor binding domain (RBD) [52]. Our analytical framework is data-driven and can be extended to study other infectious diseases.

For the limitations of this study, we have the following remarks. First, the reconstruction of *R*_*t*_ relies on the setting of the generation time (GT). We modeled the distribution of COVID-19 GT as a fixed Gamma distribution, which follows previous findings [28–32]. In a real-world situation, the time interval between the transmission generations could be variable [42, 53], which may affect the estimation of *R*_*t*_. However, the changes in *R*_*t*_ estimates due to slight variations in GT are negligible [42]. We remark that this issue is unlikely to affect our main conclusion, and our model can be extended to a more complex context with the available time-varying GT data. Second, for the *R*_*t*_ estimation parts, *C*(*t*) should be the number of COVID-19 cases onset at time *t*. However, because the data by onset are unavailable, we adopted the current dataset by reporting data as a proxy for the COVID-19 incidence time series. If one considers a constant reporting lag, the *R*_*t*_ estimates will be in exactly the same trends but shifted for the reporting lag. Considering that a similar reporting delay also occurred for the SARS-CoV-2 sequencing data, the effects of the two reporting lags may be counteracted. We note that this approximation in our analysis is unlikely to affect the conclusion in this study. In addition, with detailed reporting lag information of each individual case, adjustment for the reporting delay can surely be carried out based on our current analytical framework. Third, as a data-driven study, the estimated association should be interpreted with caution. With ecological settings, our analysis provides statistical evidence about the likelihood of causality, but the findings in this study cannot guarantee causality, which needs further biomedical experiments with a more sophisticated context.

## Conclusions

Our findings show a link between the molecular-level mutation activity of SARS-CoV-2 and population-level COVID-19 transmission to provide further evidence for a positive association between the D614G substitution and *R*_*t*_. Future studies exploring the mechanism between SARS-CoV-2 mutations and COVID-19 infectivity are warranted.

## Supplementary Information

**Additional file 1:.**

## Data Availability

All data used in this work are publicly available.

## Abbreviations

AA: Amino acid
COVID-19: Coronavirus disease 2019
D614G: amino acid substitution of aspartic acid (D) to glycine (G) on codon 614 (of the S protein of SARS-CoV-2)
GT: Generation time
LR: Likelihood ratio
GISAID: Global Initiative on Sharing All Influenza Data
MLE: Maximum likelihood estimation
RBD: Receptor binding domain
SARS-CoV-2: Severe acute respiratory syndrome coronavirus 2
SD: Standard deviation
95% CI: 95% confidence interval
